# Segmentation of Left and Right Ventricles in Cardiac MRI Using Active Contours

**DOI:** 10.1155/2017/8350680

**Published:** 2017-08-08

**Authors:** Shafiullah Soomro, Farhan Akram, Asad Munir, Chang Ha Lee, Kwang Nam Choi

**Affiliations:** ^1^Department of Computer Science and Engineering, Chung-Ang University, Seoul 156-756, Republic of Korea; ^2^Department of Computer Engineering and Mathematics, Rovira i Virgili University, 43007 Tarragona, Spain

## Abstract

Segmentation of left and right ventricles plays a crucial role in quantitatively analyzing the global and regional information in the cardiac magnetic resonance imaging (MRI). In MRI, the intensity inhomogeneity and weak or blurred object boundaries are the problems, which makes it difficult for the intensity-based segmentation methods to properly delineate the regions of interests (ROI). In this paper, a hybrid signed pressure force function (SPF) is proposed, which yields both local and global image fitted differences in an additive fashion. A characteristic term is also introduced in the SPF function to restrict the contour within the ROI. The overlapping dice index and Hausdorff-Distance metrics have been used over cardiac datasets for quantitative validation. Using 2009 LV MICCAI validation dataset, the proposed method yields DSC values of 0.95 and 0.97 for endocardial and epicardial contours, respectively. Using 2012 RV MICCAI dataset, for the endocardial region, the proposed method yields DSC values of 0.97 and 0.90 and HD values of 8.51 and 7.67 for ED and ES, respectively. For the epicardial region, it yields DSC values of 0.92 and 0.91 and HD values of 6.47 and 9.34 for ED and ES, respectively. Results show its robustness in the segmentation application of the cardiac MRI.

## 1. Introduction

Cardiac MRI is a noninvasive imaging methodology, which is used to obtain the anatomical data of a heart for clinical diagnosis of cardiovascular analysis [1]. In cardiac MRI, volumetric analysis of left ventricle (LV) and right ventricle (RV) is an initially quantified method to diagnose cardiac contractile function. A detailed understanding of the cardiac contractility is essential in the quest to prevent, diagnose, and treat heart-related disorders [2, 3]. Therefore, segmentation of LV and RV plays a crucial role in the detection and prevention of the heart attacks, which is a common cause of mortality in this century. Manual segmentation is a hectic and time-consuming job for both radiologists and cardiologists. Moreover, it can lead to a high number of false positives due to human errors, such as fatigue and distractions. Therefore, a computer-aided diagnosis (CAD) system is needed, which can assist both radiologists and cardiologists to accurately segment LV and RV boundaries in cardiac MRI.

To date, numerous segmentation methods [4–16] have been proposed to segment either one or both LV and RV. In MRI, weak or blurred edges and intensity inhomogeneities are the problems, which make it difficult for the intensity-based segmentation methods to properly extract the regions of interests (ROI). Therefore, accurate segmentation of LV and RV is still an open challenge to the researchers in the area of medical image segmentation.

Active contours are one of those methods used to segment LV and RV in cardiac MRI. Active contour model also known as snakes was introduced by Kass et al. in late 80s in which a curve is evolved towards object boundaries to delineate a region of interest [17]. Since then, active contours have been adapted abundantly in numerous image segmentation techniques. The basic idea of the active contours is to control the deformable curve and restrict it to the object boundary by minimizing a force known as a balloon force.

There are many variants of active contours, which are classified as edge-based [15–20] and region-based methods [21–30]. These methods have been contemplated for image segmentation, where their points of interest are beneficial. Edge-based methods use image gradient information to deform the level set curve towards the object boundary. However, these types of methods are not effective on images with weak edges, noise, and blurred boundaries. On the other hand, region-based models use a region-based descriptor that exploits image statistical information to evolve the contour. Among all region-based methods, Chan-Vese [23] is a broadly utilized method that is able to properly segment images with homogeneous regions. However, this method is unable to generate acceptable segmentation results in the presence of intensity inhomogeneity, which is a well-known problem in medical imaging.

A region-based active contour method proposed by Min et al. adopts an intensity-based global division algorithm [31]. In this method, two intensity means are computed for inner and two for the outer region. Therefore, it showed better performance compared to Chan-Vese method [23]. However, this method is also unable to produce acceptable segmentation results in the presence of intensity inhomogeneity. Numerous methods have proposed a viable solution to segment inhomogeneous regions by introducing image local information in their models [22–26].

A local binary fitting (LBF) method for image segmentation is proposed by Yezzi Jr. et al. in the context of intensity inhomogeneity [32]. In this method, a Gaussian kernel is introduced in the energy formulation to exploit image local information. A localized active contour method (LAC) is devised by Lankton and Tannenbaum in which global region-based methods are reformulated by replacing global means with image local information [28]. These methods can segment intensity inhomogeneous regions, unlike their global counterparts. However, the techniques explained in these methods are sensitive to the position of initial contour. Moreover, they also have high computational cost due to the complicated local information in their formulation.

Recently, hybrid methods [27–31] gained popularity among region-based methods. These methods either combine both region (local or global) and edge information or both local and global region information in their energy formulations. In [33], Zhang et al. proposed a method which combines the advantages of edge-based and region-based active contours. In this paper, a region-based signed pressure force (SPF) function is also introduced which utilizes image global intensity means from Chan-Vese method [23]. This method adapts similar approach from geodesic active contour (GAC) model. However, in their model the edge-indicator function is replaced with a region-based SPF function; moreover, the traditional regularization function is also replaced with a Gaussian smoothing. This method only uses global image intensity information; therefore, it is unable to segment intensity inhomogeneous images.

In [34], Wang et al. introduced a new energy formulation in which image local and global information from LBF and Chan-Vese methods are incorporated in an additive manner. This method is capable of handling intensity inhomogeneities and yields better segmentation results compared to the state-of-the-art methods. However, this method is sensitive to the position of initial contour. Moreover, for different types of images, the scaling constant for the additive local and global intensity distributor varies; therefore, it is very difficult to choose the correct value. Recently, Liu e al. [13] and Yang et al. [14] proposed active contour segmentation methods for cardiac MRI data. These methods segment endocardial and epicardial boundaries by using level set formulation.

In this paper, a hybrid region-based active contour method is proposed to segment left and right ventricles in a cardiac MRI. The main contribution of this work is the formulation of new SPF function, which incorporates both local and global image intensity information. Local and global intensity information is obtained from Lankton and Tannenbaum [28] and Min et al. [31] methods, respectively. Global intensity term in the proposed SPF function helps to segment objects with big intensity difference and the local intensity term plays its role to segment objects with intensity inhomogeneity. The integration of local and global intensities has overcome the limitations of Lankton and Tannenbaum and Min et al. methods, that is, sensitivity towards the initial position of contour and inability to segment intensity inhomogeneous regions. In the proposed method, a Gaussian kernel is also used to regularize the level set and eliminate the need of expensive reinitialization.

**Figure 1:**
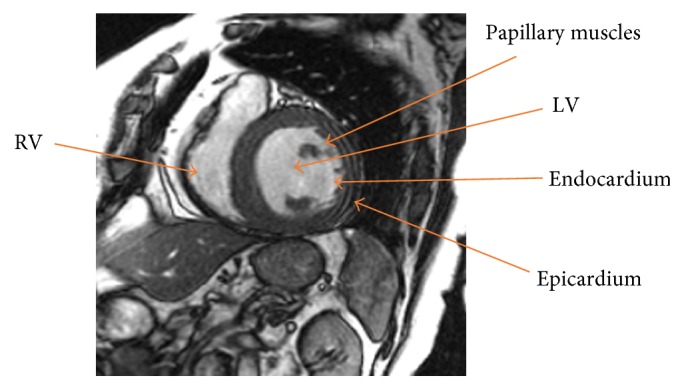
Region of interest in short axis cardiac MRI.

In order to understand the qualitative results discussed in the result section, it is necessary to understand the physical structure of both ventricles inside cardiac MRI. In short axis view of heart MRI, the myocardium is the dark area between two concentric circles surrounded by LV and RV.  Figure 1 shows LV and RV along with other important regions in cardiac MRI. Epicardium is a wall between myocardium, surrounded organs, and tissues. Endocardium wall covers the LV and RV cavity. In the presence of grey level blood flow and intensity inhomogeneity, segmentation of endocardium is a quite challenging task for both cardiologists and radiologists to quantify the cardiac contractile function. In this paper, a segmentation method is proposed which is able to segment intensity inhomogeneous regions and delineate weak object boundaries. Consequently, it helps to segment endocardium and epicardium walls for both left and right ventricles.

This paper is organized as follows. Background and previous methods are briefly discussed in Section 2. The main idea and formulation of the proposed method are presented in Section 3. Experimental results and comparisons are shown in Section 4. Quantitative analysis with the state-of-the-art methods is discussed in Section 5. Finally, conclusion and future work are given in Section 6.

## 2. Related Work and Background

### 2.1. Chan-Vese Method

Chan and Vese [23] proposed a region-based active contour method based on Mumford-Shah [22]. Let *I* : *Ω* ⊂ *R*^2^ be an input image, *ϕ* : *Ω* ⊂ *R*^2^ a level set function, and *C* a closed curve corresponding to the zero level set: *C* = {*x* ∈ *Ω*∣*ϕ*(*x*) = 0}. The Chan-Vese formulation is defined as follows:

(1) ECVC,c1,c2=λ1∫ΩIx−c12Hεϕxdx+λ2∫ΩIx−c221−Hεϕxdx+μLC+AinC,

where *L*(*C*) represents the length of the curve, which is used to regularize the contour *C*. *A*(in(*C*)) represents the area inside the curve *C*. *c*_1_, *c*_2_ are image intensities inside and outside of the curve *C*. *μ* ≥ 0, *v* ≥ 0, and (*λ*_1_, *λ*_2_) > 0 are scaling constants and *H*_*ε*_(*ϕ*) is the regularized Heaviside function which is defined as

(2) $$\begin{array}{c} H_{\varepsilon }\left(\;\phi \;\right)=\frac{1}{2}\left(\;1+\frac{2}{\pi }\arctan\left(\;\frac{\phi }{\varepsilon }\;\right)\;\right), \end{array}$$

where constant *ε* controls the smoothness of the Heaviside function. By minimizing (1) with respect to *c*_1_, *c*_2_, and *ϕ* using the steepest gradient descent [35] the following definitions and solution equations are acquired:

(3) c1=∫ΩIxHεϕxdx∫ΩHεϕxdx,

(4) c2=∫ΩIx1−Hεϕxdx∫Ω1−Hεϕxdx,

(5) ∂ϕ∂t=−λ1I−c12+λ2I−c22+μ div∇ϕ∇ϕ−vδεϕ.

In (5), *δ*_*ε*_(*ϕ*) is a smooth version of the Dirac delta function, which is defined as

(6) $$\begin{array}{c} \delta_{\varepsilon }\left(\;\phi \;\right)=\frac{\varepsilon }{\pi \left(\phi^{2}+\varepsilon^{2}\right)}, \end{array}$$

where constant *ε* controls the width of the Dirac delta function. This method is widely used to segment the images with uniform intensity distribution. However, it cannot properly segment images with intensity inhomogeneity.

### 2.2. Min et al. Method

In [31], a region-based active contour method with modified global region term is proposed to solve complicated intensity difference problem in Chan-Vese method. The proposed energy function computes two intensity means (big and small) for inside and two for outside of curve *C*. This extra information from new means provides better segmentation experience compared to Chan-Vese method. However, this method is sensitive to noise. Their energy functional based on a novel region-based term is defined as follows:

(7) EMINϕ,c1,c2,d11,d12,d21,d22=∫ΩHεIx−c1·Ix−d112Hεϕxdx+∫Ω1−HεIx−c1Ix−d122·Hεϕxdx+∫ΩHεIx−c2·Ix−d2121−Hεϕxdx+∫Ω1−HεIx−c2Ix−d222·1−Hεϕxdx,

where *H*_*ε*_(*I*(*x*) − *c*_*i*_) is a division function based on the intensity means from both inside and outside of object at *i* = 1,2. By minimizing (7) with respect to *d*_11_, *d*_12_, *d*_21_, and *d*_22_, using the steepest gradient descent [35], the following definitions are acquired:

(8) d11=∫ΩHεIx−c1IxHϵϕxdx∫ΩHεIx−c1Hϵϕxdx,

(9) d12=∫Ω1−HεIx−c1IxHϵϕxdx∫Ω1−HεIx−c1Hϵϕxdx,

(10) d21=∫ΩHεIx−c2Ix1−Hϵϕxdx∫ΩHεIx−c21−Hϵϕxdx,

(11) d22=∫Ω1−HεIx−c2Ix1−Hϵϕxdx∫Ω1−HεIx−c21−Hϵϕxdx,

where *d*_11_ and *d*_12_ are big and small intensity means inside the contour. Similarly, *d*_21_ and *d*_22_ represent the big and small intensity means outside the contour.

**Figure 2:**
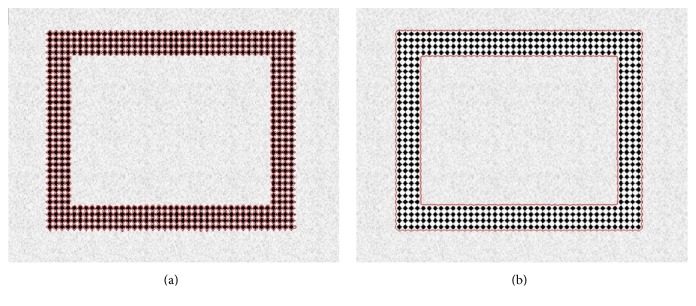
Segmentation of a complicated (texture like) region. (a) Segmentation result of the Chan-Vese method and (b) segmentation result of Min et al. method.

**Figure 3:**
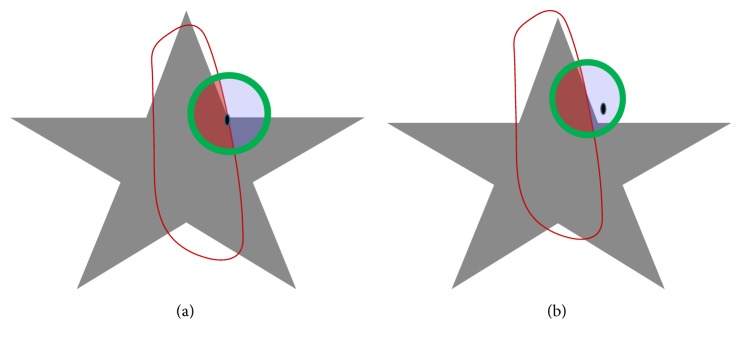
Process of taking local information, where green circle is a circular mask. (a) is a local interior region; (b) shows local exterior regions and the small dot is point *x*.

By using four values of intensity means, unlike Chan-Vese method, this method fairly improves segmentation accuracy.  Figure 2 shows segmentation of a complicated (texture like) region using both Chan-Vese [23] and Min et al. [31] methods.  Figure 3(a) shows that Chan-Vese method produces an unacceptable segmentation result. This method takes small black dots as separate regions that lead to an unacceptable segmentation of the big rectangular region, which is the actual region of interest. In turn, Min et al. method is able to properly segment the rectangular region as shown in Figure 2(b).

### 2.3. Zhang et al. Method

Primarily an edge-indicator function was proposed in GAC method [19] to segment an object by evolving the level set curve towards object boundaries. However, this method was not able to segment global structure of the object. In [26], Li et al. proposed a region-based segmentation method, which combines the advantages of GAC and Chan-Vese methods. In this method, a region-based signed pressure force (SPF) function is introduced to replace the edge-indicator function in GAC method. SPF function helps to segment global structure of the given image using intensity means from inside and outside of the curve. A Gaussian kernel is used to regularize the level set, which also removes the need of its reinitialization. Let *I* : *Ω* ⊂ *R*^2^ be the given image and *ϕ* : *Ω* ⊂ *R*^2^ a level set curve; then the solution to their energy functional is defined as

(12) $$\begin{array}{c} \frac{\partial \phi }{\partial t}=spf\left(\;I\left(\;x\;\right)\;\right)\cdot \alpha \left|\;\nabla \phi \;\right|, \end{array}$$

where *α* > 0 is a scaling parameter and spf(*I*) is the SPF function, which is defined as

(13) $$\begin{array}{c} spf\left(\;I\;\right)=\frac{I\left(x\right)-\left(c_{1}+c_{2}\right)/2}{\max\left(\left|\left(x\right)-\left(c_{1}+c_{2}\right)/2\right|\right)}. \end{array}$$

In (13), the value of the SPF function is in the range [−1,1]. It shrinks the contour when it is defined outside and expands when defined inside of the object. *c*_1_ and *c*_2_ are the image intensity means defined in (3) and (4), respectively.

### 2.4. Localized Chan-Vese Method

In [23], Chan and Vese proposed a local active contour method in which different well-known global active contour models are remodeled by replacing the global statistical information in their energy functionals with the local ones. This method can reformulate any global region-based model to be mimicked as a local model. Let *I* : *Ω* ⊂ *R*^2^ be the given image and *C* be a closed curve corresponding to zero level set: *C* = {*x* ∈ *Ω*∣*ϕ*(*x*) = 0}. The energy functional is defined as follows:

(14) ELCV=∫Ωxδεϕx∫ΩyBx,y·FIy,ϕydy dx+λ∫Ωx∇Hεϕxdx,

where *H*_*ε*_(*ϕ*) and *δ*_*ε*_(*ϕ*) are smooth versions of Heaviside and Dirac delta functions, defined in (2) and (6). *B*(*x*, *y*) is a mask function which helps to detect the local regions in terms of radius *r* defined as follows:

(15) $$\begin{array}{c} B\left(\;x,y\;\right)=\left\{\begin{array}{cc} 1, & \left\|x-y\right\|<r,\\ 0, & otherwise. \end{array}\right. \end{array}$$

*B*(*x*, *y*) will be 1 when point *y* is within the mask of radius *r* centered at *x*; otherwise, it will be 0. In Figure 3, the local neighborhood is shown in green and the contour is shown in red. The process to compute the local intensity information for both regions inside and outside of the contour is shown in Figures 3(a) and 3(b), respectively.

The energy function *F*(*I*(*y*), *ϕ*(*x*)) is reformulated by replacing the global means *c*_1_ and *c*_2_ with local means *u*_1_ and *u*_2_, as follows:

(16) FIy,ϕx=HεϕyIy−u12+1−HεϕyIy−u22.

By substituting *F*(*I*(*y*), *ϕ*(*x*)) in (14) following localized energy function is formulated:

(17) ELCV=∫Ωxδεϕx∫ΩxBx,y·HεϕyIy−u12+1−HεϕyIy−u12dy dx+λ∫Ωx∇Hεϕxdx,

where *u*_1_ and *u*_2_ are the local intensity means inside and outside of the contour, respectively. These intensities localized by mask function *B*(*x*, *y*) at point *x* are defined as

(18) u1=∫ΩyBx,yIyHεϕydy∫ΩyBx,yHεϕydy

(19) u2=∫ΩyBx,yIy1−Hεϕydy∫ΩyBx,y1−Hεϕydy.

This method is capable of segmenting images with intensity inhomogeneities. However, it is sensitive to the initial position of the contour.

## 3. The Proposed Method

In this paper, a novel region-based active contour method is formulated, which uses both local and global image statistical information. An energy functional is devised using a new region-based SPF function based on Lankton and Tannenbaum [28] and Min et al. [31] intensity terms, which drives the zero level set curve towards the object boundary. During the curve evolution, its movement depends on the sign of the SPF function, which moves inwards if SPF is positive and outwards if it is negative. Let *I* : *Ω* ⊂ *R*^2^ be the given image and *ϕ* : *Ω* ⊂ *R*^2^ a level set curve; then the proposed energy functional is defined as

(20) $$\begin{array}{c} E_{LG}\left(\;\phi \;\right)=\lambda L_{LG}\left(\;\phi \;\right)+vA_{LG}\left(\;\phi \;\right), \end{array}$$

where *v* ≥ 0 and *λ* > 0 are the scaling constants. *L*_*LG*_(*ϕ*) is a length and *A*_*LG*_(*ϕ*) is an area term, defined as

(21) LLGϕ=∫ΩspfLGIδεϕ∇ϕdx,ALGϕ=∫ΩspfLGIHε−ϕdx.

In the above equations, *H*_*ε*_(*ϕ*) and *δ*_*ε*_(*ϕ*) are smooth versions of the Heaviside and Dirac delta functions, which are defined in (2) and (6), respectively. The energy *A*_*LG*_(*ϕ*) controls the inner force, which computes the region information inside and outside of the curve and evolves the curve towards the object boundary. In turn, *L*_*LG*_(*ϕ*) term deals with the curvature of the object boundary and regularizes the curve. spf_*LG*_(*I*) is the proposed SPF function, which incorporates local and global characteristics from Kass et al. [17] and Lankton and Tannenbaum [28] methods, respectively. Like a traditional SPF function, the value of the proposed SPF function is in the range [1, −1]. The only difference is that it uses both local and global intensity means in its formulation. The proposed SPF function is defined as

(22) $$\begin{array}{c} {spf}_{LG}\left(\;I\;\right)=w\left(\;{spf}_{G}\left(\;I\;\right)\;\right)+\left(\;1-w\;\right){spf}_{L}\left(\;I\;\right), \end{array}$$

where spf_*G*_(*I*) and spf_*L*_(*I*) are global and local SPF functions, respectively. *w* is a scaling constant whose value lies in 0 ≤ *w* ≤ 1. It decides which SPF term will play a key role during the contour evolution. When *w* is close to 0 then spf_*L*_(*I*) with the local terms will be dominant. In turn, when *w* is close to 1 then spf_*G*_(*I*) with the global terms will be dominant. Selection of the parameter *w* depends on the type of image used for the segmentation. If the given image has a uniform intensity distribution then *w* should be close to 1. In turn, if the given image has intensity inhomogeneity then *w* should be close to 0. The global SPF function spf_*G*_(*I*) used in (22) is defined as follows:

(23) $$\begin{array}{c} {spf}_{G}\left(\;I\;\right)=\frac{\left(I\left(x\right)-I_{GFI}\right)M^{k}}{\max\left(\left|I\left(x\right)-I_{GFI}\right|\right)}, \end{array}$$

where *I*_GFI_ is a global fitted image defined as

(24) IGFI=d11+d12Hεϕx+d21+d221−Hεϕx,

where *d*_11_, *d*_12_, *d*_21_, and *d*_22_ are the global intensity means, which are defined in (8), (9), (10), and (11), respectively. Similarly, the local SPF spf_*L*_(*I*) used in (22) is defined as

(25) $$\begin{array}{c} {spf}_{L}\left(\;I\;\right)=\frac{\left(I\left(x\right)-\left(u_{1}+u_{2}\right)/2\right)M^{k}}{\max\left(\left|I\left(x\right)-\left(u_{1}+u_{2}\right)/2\right|\right)}, \end{array}$$

where *u*_1_ and *u*_2_ are the local intensity means, which are defined in (18) and (19), respectively. In (23) and (25), a characteristic term *M*^*k*^ is used to restrict the evolution of the contour inwards (towards the region of interest), which is defined as

(26) $$\begin{array}{c} M^{k}\left(\;x\;\right)=\left\{\begin{array}{cc} \Omega ⟶1, & k=0\\ \phi \left(x\right)>0, & k\neq 0, \end{array}\right. \end{array}$$

where *k* is a nonnegative integer. By minimizing (20) with respect to *ϕ* using the steepest gradient descent [30], the following solution is obtained:

(27) ∂ϕ∂t=δεϕλ divspfLGI·∇ϕ∇ϕ+v spfLGI,

where spf_*LG*_(*I*) is the proposed SPF function which incorporates both local and global intensity information in an additive manner as defined in (22).

In the traditional level set methods [12, 14, 15], the level set function is reinitialized after each time step for smooth transitions during the curve evolution process. In [13], an energy penalization term is introduced by Liu et al., which maintains the level set function as a signed distance function (SDF) to remove the need of reinitialization. In this paper, a Gaussian kernel is used, which not only regularizes the level set curve but also removes the computationally expensive reinitialization step. For outmoded level set methods, it is essential to initialize level set function as a signed distance function (SDF). The initial level set function is defined as

(28) $$\begin{array}{c} \phi \left(\;x,t=0\;\right)=\left\{\begin{array}{cc} -\rho & x\in \Omega_{0}-\partial \Omega_{0}\\ 0 & x\in \partial \Omega_{0}\\ \rho & x\in \Omega -\Omega_{0}, \end{array}\right. \end{array}$$

where *ρ* > 0 is a constant (in this paper *ρ* = 2). Finally, the iterative steps of the proposed method are summarized as follows:

1. Initialize the level set function *ϕ* using *ϕ*(*x*, *t* = 0) from (28).
2. Initialize the characteristic term *M*^*k*^ using *M*^0^ from (25).
3. Compute *d*_11_, *d*_12_, *d*_21_, *d*_22_, *u*_1_, and *u*_2_ using (8), (9), (10), (11), (18), and (19), respectively.
4. Compute *M*^*k*^ from (26) and spf_*LG*_(*I*) using (22).
5. Solve the partial differential equation for *ϕ* using (27).
6. Stop if the level set function from the solution is stationary. Otherwise, go to step (3) and iterate.

## 4. Experimental Results

The proposed method is validated on 15 training and 15 validation datasets from the MICCAI 2009 [36] for left ventricle segmentation. Using* Test 1* dataset from MICCAI 2012 [37] proposed method is also validated for right ventricle segmentation, respectively. These datasets contain cardiac MRI short axis volumetric data along with their respective ground truths. The proposed method is implemented in MATLAB 8.5 version in Windows 8 environment using 2.97 GHz Intel Core-i7 processor with 4 GB RAM.

### 4.1. Parameters Selection

For the proposed method the following parameters are used for all the experiments: *λ* = 1, *v* = 0.002 × 255 × 255, Δ*t* = 1.0, *r* = 10, *w* = 0.03, and *ε* = 1.5. It is critical to properly tune the parameter *w*, which controls the level segmentation of homogeneous and inhomogeneous regions. *w* ranges between 0 and 1; it is chosen small for intensity inhomogeneous objects while for homogenous intensity regions *w* is chosen near to 1.

**Figure 4:**
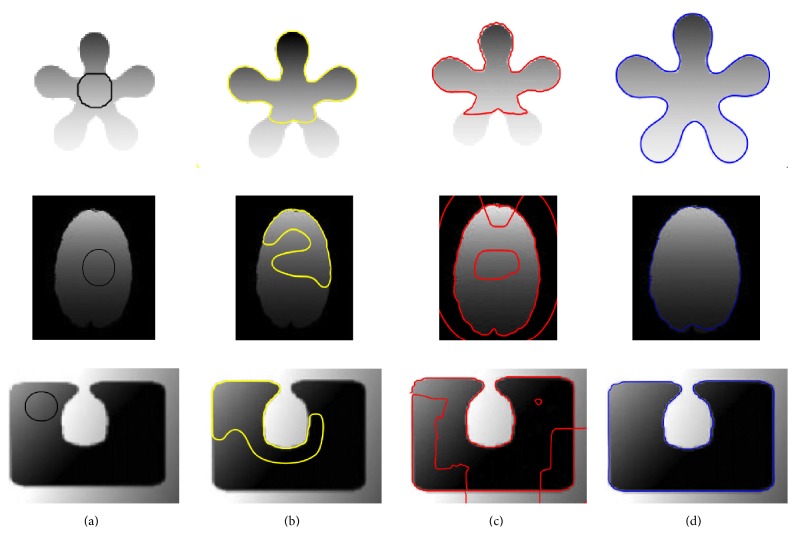
Image segmentation using synthetic images. (a) Original image with the initial contour, (b) Chan and Vese method [23], (c) LBF method [32], and (d) the proposed method.

**Figure 5:**
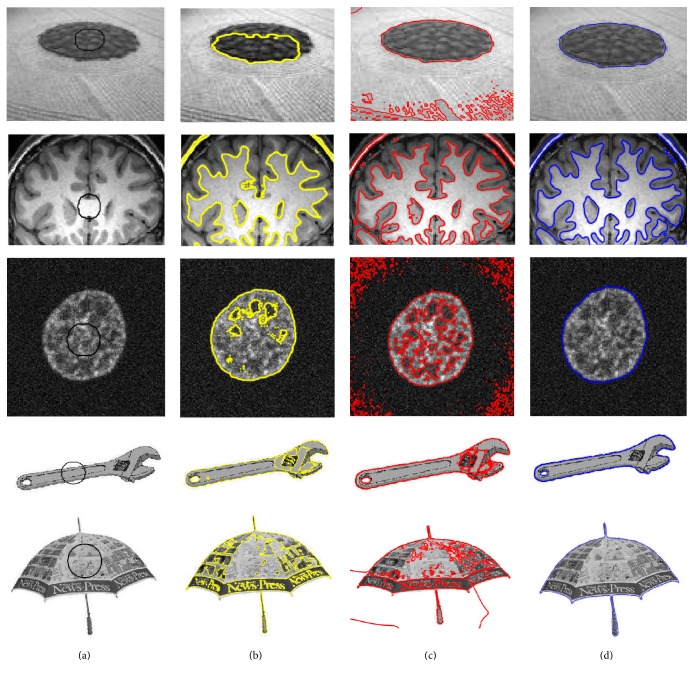
Image segmentation using real images. (a) Original image with the initial contour, (b) Chan and Vese method [23], (c) LBF method [32], and (d) the proposed method.

Initially, the proposed method has been tested on both synthetic and real images.  Figure 4 shows the qualitative segmentation comparison with the state-of-the-art using the synthetic images. Column (a) shows the original images with the initial contour, columns (b), (c), and (d) show the segmentation results using the Lankton and Tannenbaum [28], LBF [25], and proposed method, respectively. Similarly, the segmentation results using the real images are shown in Figure 5. Column (a) shows the original images with the initial contour, columns (b), (c), and (d) show the segmentation results using the Lankton and Tannenbaum [28], LBF [32], and proposed method, respectively. Results show that the proposed method yields high accuracy compared to the state-of-the-art methods, which are unable to properly segment the regions of interest.

### 4.2. Endocardium Segmentation

**Figure 6:**
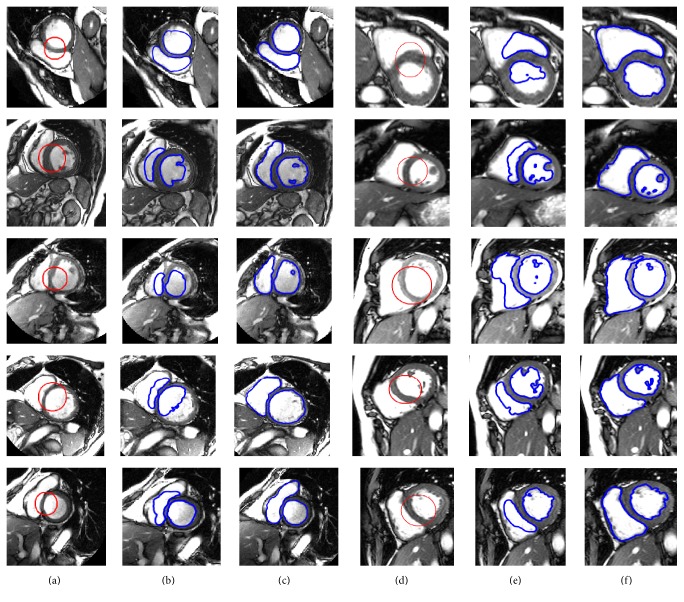
Segmentation of left and right ventricles. (a) Original images from MICCAI 2009 database [36] with the initial contour, (c) original images from MICCAI 2012 database [37] with the initial contour, ((b) and (e)) intermediate results using the proposed method, and ((c) and (f)) final segmentation results using proposed method.

The proposed method is used to delineate the endocardial boundaries of both LV and RV from cardiac MRI.  Figure 6 demonstrates the segmentation results of both left and right ventricles from the cardiac MRI using two different databases [33, 38], where columns (a) and (d) show the images with the initial contours from the MICCAI 2009 [36] and MICCAI 2012 [37] databases, respectively. Columns (b) and (e) show intermediate segmentation results using the proposed method. In turn, columns (c) and (f) show final segmentation results using the proposed method.

**Figure 7:**
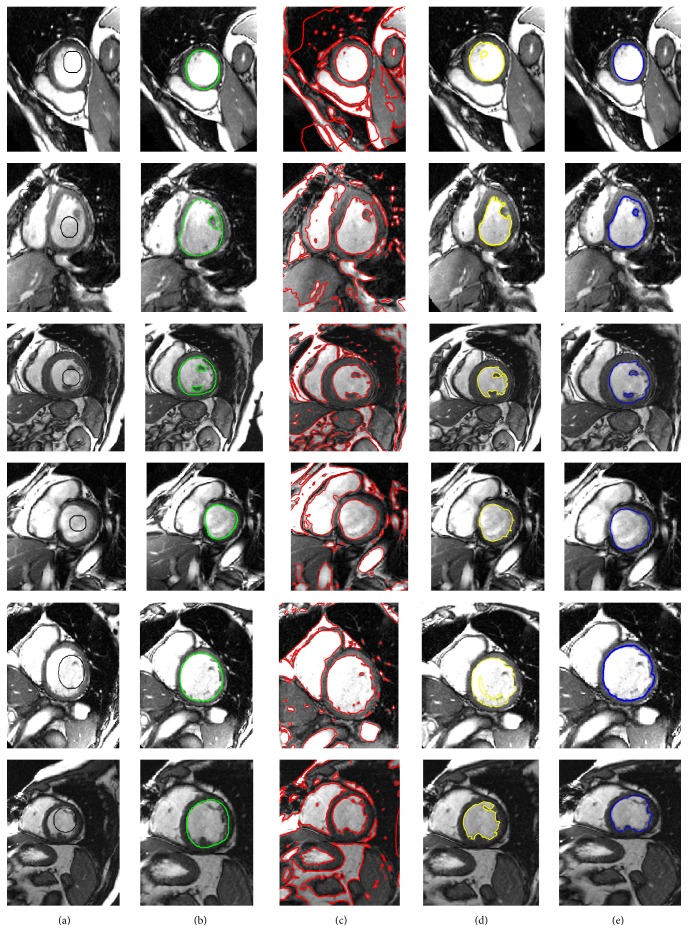
Segmentation of LV using MICCAI 2009 database [36]. (a) Original image with the initial contour, (b) ground truth, (c) LBF method [32], (d) Chan and Vese method [23], and (e) the proposed method.

**Figure 8:**
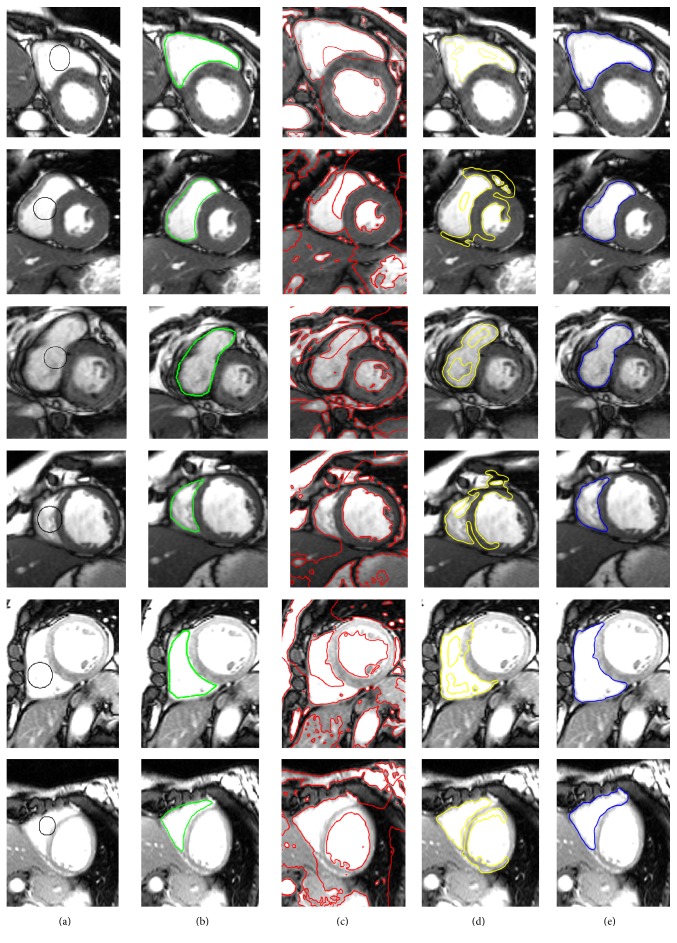
Segmentation of RV using MICCAI 2012 database [37]. (a) Original image with the initial contour, (b) ground truth, (c) LBF method [25], (d) Lankton and Tannenbaum method [28], and (e) the proposed method.

Figure 7 shows the left ventricle segmentation using the cardiac MRI from the MICCAI 2009 [36] database. Similarly, Figure 8 shows the right ventricle segmentation using the cardiac MRI from the MICCAI 2012 [37] database. The qualitative comparison of the segmentation results of the proposed method with the LBF [25] and Lankton and Tannenbaum [28] methods is also shown in Figures 7 and 8. In Figures 7 and 8, column (a) shows the original images with the initial contour, column (b) shows the ground truths, columns (c), (d), and (e) show segmentation results of the LBF [25], Lankton and Tannenbaum [28], and proposed method, respectively. It is evident from the results that the proposed method yields better segmentation than both Lankton and Tannenbaum and LBF methods.

### 4.3. Epicardium Segmentation

**Figure 9:**
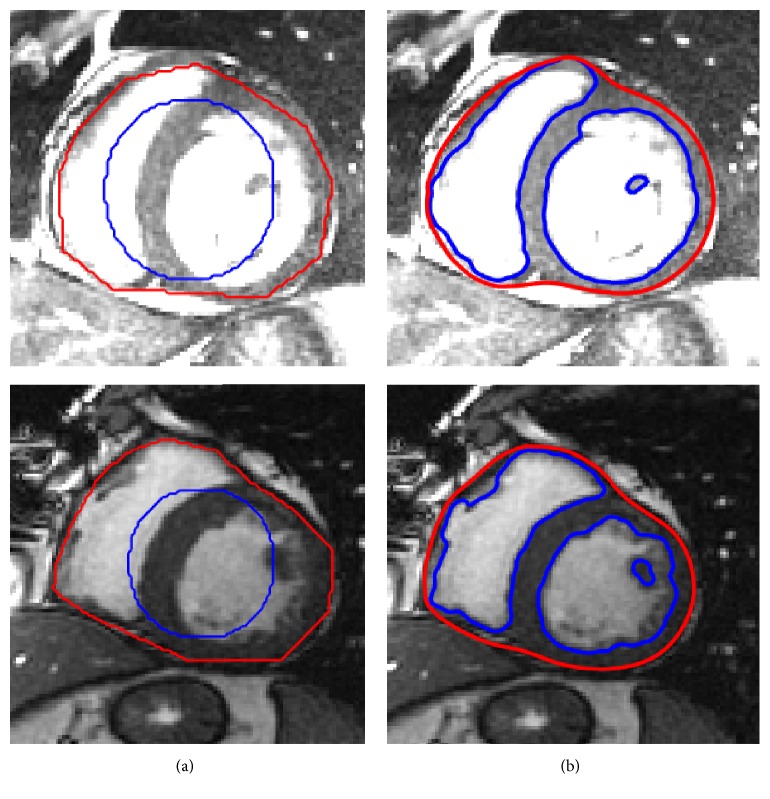
Segmentation of endocardial and epicardial contours using proposed method. (a) Initialization contours. (b) Final segmentation result.

**Figure 10:**
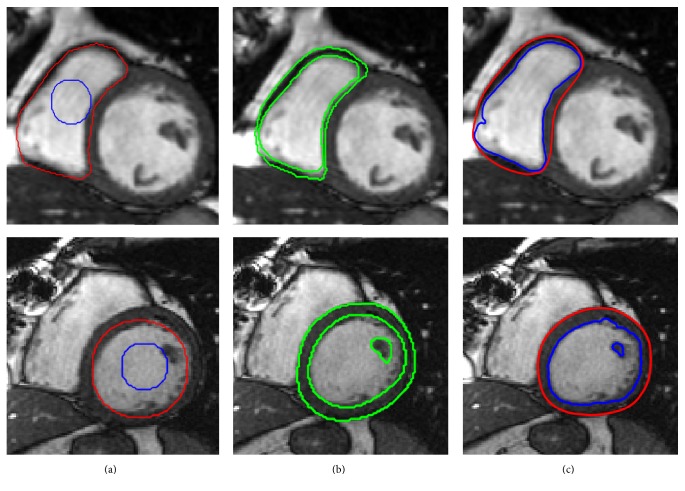
Segmentation endocardial and epicardial contours using proposed method. (a) Initialization contours, (b) ground truth, and (c) final segmentation result.

The proposed method is also used to segment epicardial contours of both ventricles (LV and RV). In [13], Liu et al. adapted the anatomical knowledge of the heart in his model and proposed a two-step method, where both epicardial and endocardial contours are represented by a single level set function. Inspired by the work of Liu et al. [13], the proposed method can also segment epicardial and endocardial contours at the same time but, instead of two steps, it can properly delineate both contours (ED and EP) in single step level set evolution.  Figure 9 explains the complete segmentation process. The contour is initialized with two circular level sets manually as shown in Figure 9(a), one inside ventricles and one near the endocardial boundary. After the evolution process, the final contours (epicardial and endocardial) will capture both RV and LV as shown in Figure 9(b). Furthermore, more experiments are also performed on single ventricle in order to extract epicardial and endocardial contours.  Figure 10 illustrates the process of single ventricle segmentation, where Figure 10(a) shows the initial contours, Figure 10(b) shows the ground truth, and Figure 10(c) shows the final segmentation result using proposed method.

## 5. Discussion

### 5.1. Quantitative Analysis

Dice coefficient (DSC) and Hausdorff-Distance (HD) metrics are used for the quantitative analysis and comparison with the state-of-the-art methods. DSC measures how well segmentation *S* overlaps the ground truth *G*. Segmentation results have high accuracy when DSC value is close to 1. DSC is defined as

(29) $$\begin{array}{c} DSC\left(\;G,S\;\right)=\frac{2\left|\Omega_{G}\cap \Omega_{S}\right|}{\left|\Omega_{G}\right|+\left|\Omega_{S}\right|}, \end{array}$$

where *Ω*_*G*_ is the ground truth region and *Ω*_*S*_ is the segmented region. HD is the second statistical measure used for the quantitative evaluation in this paper. It is the distance between the segmented region and the ground truth contour. Segmentation results have high accuracy when the HD value is close to 0. HD is defined as

(30) HDG,S=max⁡maxi⁡dgi,S,maxj⁡dsj,G,

where the ground truth *G* and the segmented region *S* contain a group of points *G* = {*g*_1_, *g*_2_, *g*_3_,…, *g*_*n*_} and *S* = {*s*_1_, *s*_2_, *s*_3_,…, *s*_*n*_}, respectively, and *d* is the distance from *g*_*i*_ to the nearest point on the region *S*.

**Figure 11:**
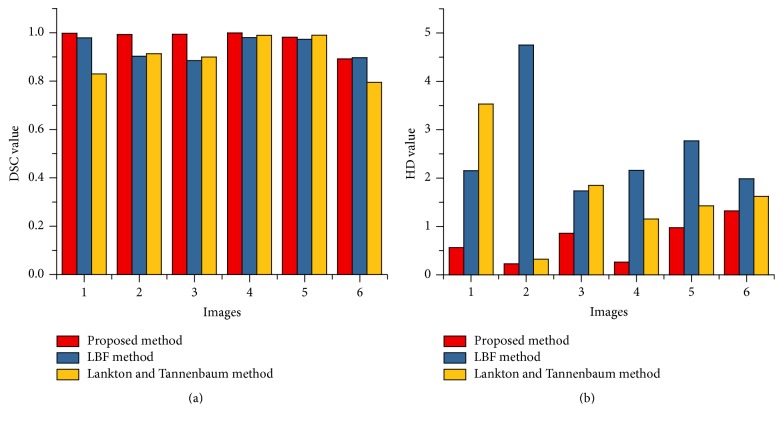
DSC (a) and HD (b) values for LV cardiac MRI segmentation based on Figure 7.

**Figure 12:**
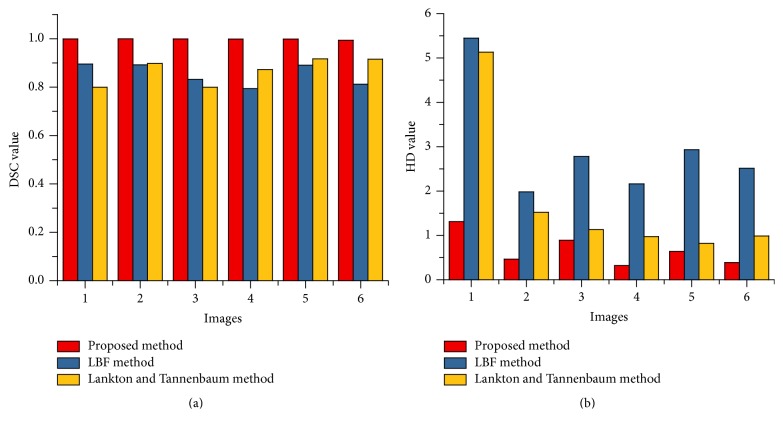
DSC (a) and HD (b) values for LV cardiac MRI segmentation based on Figure 8.

Figure 11 shows DSC and HD value of LV cardiac MRI segmentation based on Figure 7. Similarly, Figure 12 shows the DSC and HD value of RV cardiac MRI segmentation based on Figure 8. It shows that proposed method gets minimum HD values and highest DSC value compared to other intensity-based methods.

The segmentation accuracy of the proposed method is being validated with cardiac application based methods using DSC and HD metrics as shown in Table 1 using the training dataset from MICCAI 2009 [36].  Table 2 shows a segmentation accuracy comparison between the proposed method and the state of the art using DSC metrics on validation dataset from MICCAI 2009 [36]. It shows that the proposed method yields highest DSC value among all methods, which means the segmentation results of proposed method are closest to their respective ground truths. In Table 3, cardiac MRI MICCAI 2012 [37] test dataset is used to compute DSC and HD metrics to compare the accuracy of the proposed method with the state of the art. It shows that the proposed method yields better segmentation accuracy compared to previous methods.

### 5.2. Selection of Parameters *w* and *r*

Parameter *w* plays an essential role in the proposed segmentation method. This parameter deals with the measure of local and global intensity force during the segmentation process. The local SPF force is scaled with a (1 − *w*) parameter and the global SPF force is scaled with *w* parameter, where 0 ≤ *w* ≤ 1. When the input image is affected by the intensity inhomogeneity, the value decided for *w* should be near 0 to diminish the interference of global SPF. Similarly, if the image has uniform intensity distribution and it is affected by the noise, then the selected value of *w* should be near 1 to make global SPF force dominant.

The parameter *r* > 0 plays an important role in segmenting local structure of an image. If the image has one or more objects with complex structures, then a small value of *r* is chosen. In turn, for images with large objects and fairly simple structure, big value of *r* is chosen. In this paper, for the images which have the objects with complex structures, the chosen value of *r* is between 4 and 8. In turn, for large objects, a value between 12 and 20 is chosen.

## 6. Conclusion

In this paper, a new region-based active contour method is presented to segment both left and right ventricles in cardiac MRI. The energy functional is based on a new region-based SPF function, which is formulated using both local and global statistical information in an additive manner. A selection parameter *w* plays an important role in switching between local and global SPF functions. The local part of the SPF function helps to accurately segment intensity inhomogeneous images. In turn, the global part of SPF function helps to segment homogeneous images with or without noise. The global intensity mean from Min et al. method is used to construct global SPF function. It helps to segment complex intensity regions which are not segmented by traditional global region-based active contours like Chan-Vese method.

Finally, a Gaussian kernel is used to regularize the level set curve after each time step, which also eliminates the need of expensive reinitialization. Experimental results demonstrate that the proposed method can efficiently segment LV and RV separately or together in cardiac MRI. Furthermore, a quantitative comparison of the proposed method with the state-of-the-art methods demonstrates that the proposed method yields better segmentation accuracy.

## Figures and Tables

**Table 1:** Results of training dataset for 2009 LV MICCAI [36].

Patient	HD		DSC	
	Endo	Epi	Endo	Epi
SC-HF-I-5	1.4425	1.0095	0.9942	0.97
SC-HF-I-6	0.2578	0.2686	0.9923	0.9985
SC-HF-I-7	0.3712	0.4221	0.994	0.9902
SC-HF-I-8	0.3833	0.3869	0.997	0.9908
SC-HF-NI-7	0.9954	1.035	0.9923	0.9951
SC-HF-NI-11	0.6446	0.5569	0.9843	0.9915
SC-HF-NI-31	0.438	0.3719	0.9884	0.9922
SC-HF-NI-33	0.6774	0.573	0.9894	0.9912
SC-HYP-6	0.733	0.5577	0.9846	0.9944
SC-HYP-7	0.9363	1.2184	0.979	0.9707
SC-HYP-8	0.4615	0.5653	0.9936	0.9885
SC-HYP-37	0.6303	0.6469	0.994	0.9872
SC-N-5	1.0042	0.5513	0.9897	0.9913
SC-N-6	0.7766	0.4009	0.9863	0.991
SC-N-7	0.2243	0.8215	1	0.9886

**Table 2:** Mean (± standard deviation) of DSC metric: left ventricle segmentation results of different methods using 2009 LV MICCAI [36] validation dataset.

Group	[39]	[40]	[41]	[13]	Proposed method
DSC (endo)	0.89 ± 0.03	0.89 ± 0.03	0.89 ± 0.04	0.92 ± 0.03	0.95 ± 0.03
DSC (epi)	0.94 ± 0.02	0.93 ± 0.03	0.92 ± 0.02	0.95 ± 0.01	0.97 ± 0.01

**Table 3:** Mean (± standard deviation) of DSC and HD metrics: right ventricle segmentation results of different methods averaged over ED (end-diastole) and ES (end-systole) using 2012 RV MICCAI [37] test dataset.

	[42]		[10]		[13]		Proposed method	
	ED	ES	ED	ES	ED	ES	ED	ES
Endo (DM)	0.86 (0.11)	0.69 (0.11)	0.88 (0.11)	0.77 (0.18)	0.90 (0.15)	0.82 (0.13)	0.97 (0.09)	0.90 (0.10)
Endo (HD mm)	7.70 (3.74)	11.16 (5.53)	7.69 (6.03)	10.71 (7.69)	7.51 (5.47)	10.50 (8.03)	8.51 (6.83)	7.67 (5.36)
Epi (DM)	0.88 (0.08)	0.77 (0.17)	0.90 (0.08)	0.82 (0.13)	0.89 (0.08)	0.83 (0.12)	0.92 (0.07)	0.91 (0.11)
Epi (HD mm)	7.93 (3.72)	11.72 (5.44)	8.02 (5.96)	11.52 (7.70)	9.36 (8.19)	12.58 (9.03)	6.47 (4.32)	9.34 (6.69)
